# Extremely low nucleotide polymorphism in *Pinus krempfii* Lecomte, a unique flat needle pine endemic to Vietnam

**DOI:** 10.1002/ece3.1091

**Published:** 2014-05-08

**Authors:** Baosheng Wang, Marjan Khalili Mahani, Wei Lun Ng, Junko Kusumi, Hai Hong Phi, Nobuyuki Inomata, Xiao-Ru Wang, Alfred E Szmidt

**Affiliations:** 1 Department of Ecology and Environmental Science, Umeå UniversityUmeå, Sweden; 2 Department of Biology, Kyushu UniversityFukuoka, Japan; 3 Vietnamese Academy of Forest SciencesHanoi, Vietnam; 4 Department of Environmental Science, International College of Arts and Sciences, Fukuoka Women's UniversityFukuoka, Japan

**Keywords:** DNA polymorphism, genetic diversity, *Pinus krempfii*, population history

## Abstract

*Pinus krempfii* Lecomte is a morphologically and ecologically unique pine, endemic to Vietnam. It is regarded as vulnerable species with distribution limited to just two provinces: Khanh Hoa and Lam Dong. Although a few phylogenetic studies have included this species, almost nothing is known about its genetic features. In particular, there are no studies addressing the levels and patterns of genetic variation in natural populations of *P. krempfii*. In this study, we sampled 57 individuals from six natural populations of *P. krempfii* and analyzed their sequence variation in ten nuclear gene regions (approximately 9 kb) and 14 mitochondrial (*mt*) DNA regions (approximately 10 kb). We also analyzed variation at seven chloroplast (*cp*) microsatellite (SSR) loci. We found very low haplotype and nucleotide diversity at nuclear loci compared with other pine species. Furthermore, all investigated populations were monomorphic across all mitochondrial DNA (*mt*DNA) regions included in our study, which are polymorphic in other pine species. Population differentiation at nuclear loci was low (5.2%) but significant. However, structure analysis of nuclear loci did not detect genetically differentiated groups of populations. Approximate Bayesian computation (ABC) using nuclear sequence data and mismatch distribution analysis for *cp*SSR loci suggested recent expansion of the species. The implications of these findings for the management and conservation of *P. krempfii* genetic resources were discussed.

## Introduction

**Figure 1:**
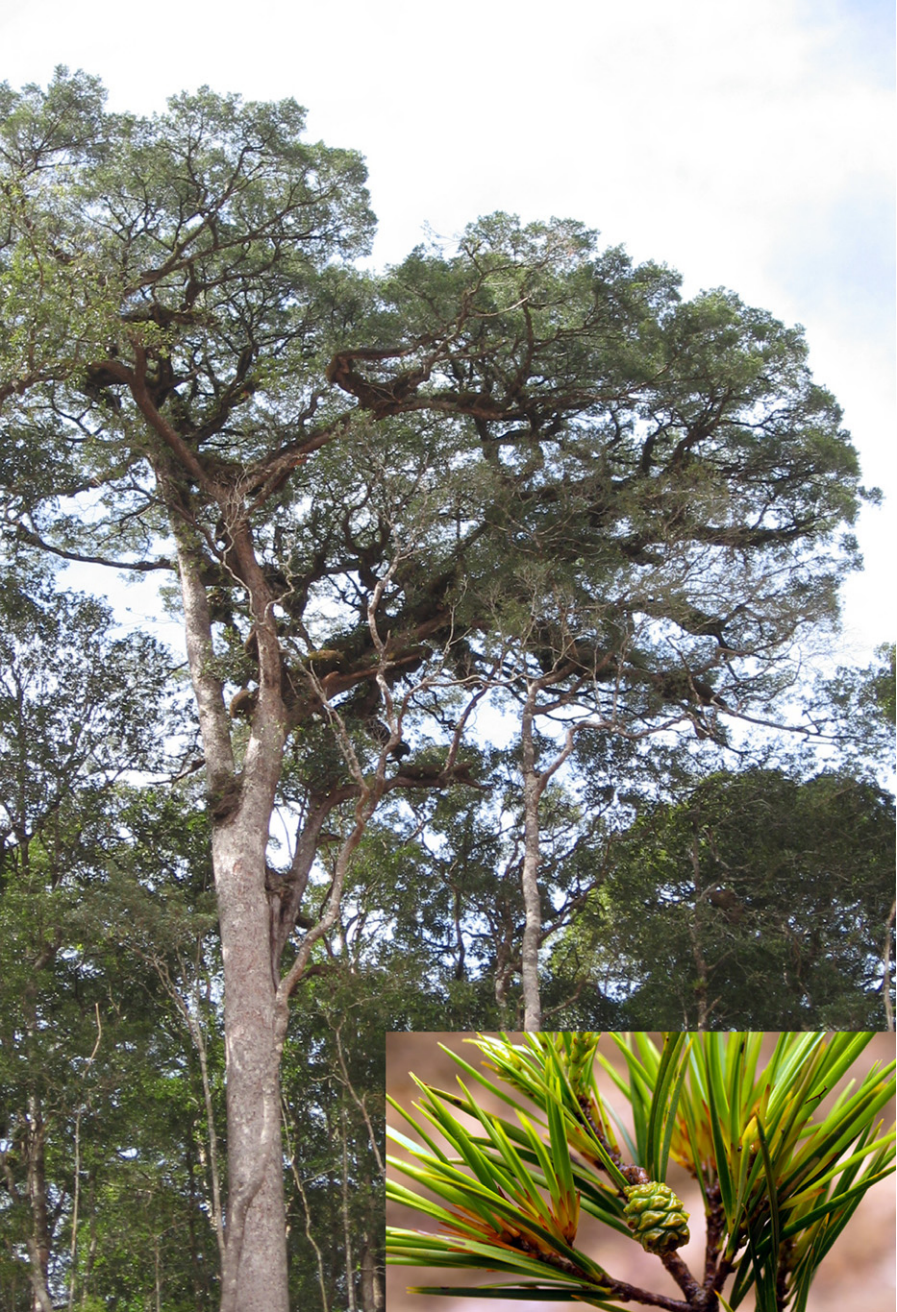
Adult tree and twigs with cone of *Pinus krempfii* in Lam Dong province, Vietnam. Photographs by Tran Tien.

*Pinus krempfii* Lecomte (Syn: *Ducampopinus krempfii* (Lecomte) A. Chev.) is a unique pine, endemic to Vietnam. It is canopy emergent tree (up to 40 m tall) usually occurring at steep slopes at elevations of 1200–2000 m (Fig. 1; Nguyen and Thomas 2004). On morphological, anatomical, physiological, chemical, and ecological grounds, *P. krempfii* is probably the most unusual species in the genus *Pinus*. Morphologically it differs from all other pines by having two flat leaf-like needles rather than typical pine needles. As a result, since its first description in 1921 by Lecomte (1921), there has been considerable controversy over its classification (see e.g., Price et al. 1998 for more details). In most recent classifications, *P. krempfii* has been considered to belong to the subgenus *Strobus* (Price et al. 1998; Wang et al. 1999; Gernandt et al. 2005). A fossil-calibrated molecular clock study suggested that the diversification within the genus *Pinus* was relatively recent with the *P. krempfii* lineage dating back 14–27 million years ago (Willyard et al. 2007).

*Pinus krempfii* is regarded as a vulnerable species, and its distribution is limited to just two provinces in Vietnam: Khanh Hoa and Lam Dong at evaluations of 1200–2000 m (Nguyen and Thomas 2004). Its extant populations are <50 km apart from each other with total range of distribution <2000 km^2^ (Nguyen and Thomas 2004). *Pinus krempfii* occurs naturally in evergreen subtropical forests on moist soils with well-developed humus layers (Nguyen and Thomas 2004). It occurs in small populations of 10–30 trees and grows together with species of *Fagaceae*, *Magnoliaceae*, *Lauraceae*, *Cryptocarya* sp., *Illicium* sp., *Rhodoleia* sp., and *Podocarpus* sp., which form very dense forests (Nguyen 1993). Seedlings of *P. krempfii* are found under the forest canopy, but juveniles with diameter >5 cm are rare, because most are killed by fungi infections (Phi HH unpublished data). *Pinus krempfii* is shade-tolerant, and physiological study revealed that the flattened leaves of this species were adapted to function optimally under low-light conditions (Brodribb and Feild 2008). In spite of biological and ecological importance of this unusual species, there is no information about the levels and patterns of genetic variation in its natural populations. Genetic markers have been used to study the phylogenetic position of *P. krempfii* and have revealed its status as a member of subgenus *Strobus* (Wang et al. 1999, 2000; Willyard et al. 2007; Parks et al. 2012). However, only few individuals of *P. krempfii* have been involved in these studies, and the levels and patterns of DNA variation in its natural populations are unclear. This situation is unfortunate because information about genetic diversity is crucial for the understanding of a species’ evolution, and for devising strategies to protect and preserve its genetic resources. Such information also provides a historical perspective on evolutionary changes of a species and helps us to predict how populations will respond to future environmental changes.

In this study, we sampled six populations of *P. krempfii* across its natural distribution and surveyed nucleotide polymorphism in ten nuclear and 14 mitochondrial (*mt*) regions. We also analyzed seven chloroplast microsatellite loci (*cp*SSR). Our specific questions were: (1) What are the levels and patterns of DNA polymorphism in *P. krempfii* compared to other pine species? (2) Is there strong genetic structure in this isolated subtropical pine? (3) What can be learned about its demographic history? This investigation is the first effort toward understanding population genetic features of this unique pine, and will be a valuable reference for its conservation.

## Materials and Methods

### Plant materials

**Figure 2:**
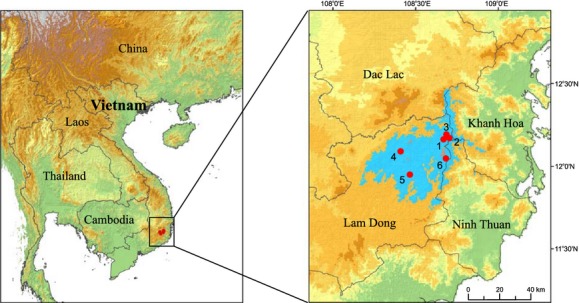
The present-day distribution (light blue) of *Pinus krempfii* in Vietnam and geographical location of six sampled populations (red dots, 1–6) from Lam Dong province. The distances separating sampled populations are <50 km.

The distances separating individual populations of *P. krempfii* are <50 km, and each population has fewer than 30 mature trees (Nguyen and Thomas 2004). In this study, we sampled six representative populations from three regions of the natural distribution of *P. krempfii*: Da Chay (Nos. 1–3), Cong Troi (Nos. 4–5), and Bidoup (No. 6; Fig. 2). The names, locations, and sample sizes of the investigated populations are listed in Table 1. For each population, needles were collected from 9 to 13 mature trees that were at least 50 m apart, except for the population Cong Troi 102, where only three mature individuals were found. In addition, one individual of *Pinus parviflora* (Siebold & Zucc.) was sampled at the Kyushu University campus and used as outgroup. Needles were dried and preserved in silica gel until DNA extraction.

**Table 1:** Geographic locations, sample sizes (*N*), the number of segregating sites (*S*), nucleotide polymorphism (*θ*_*w*_, total sites; *θ*_*ws*_ silent sites), nucleotide diversity (*π*_*t*_, total sites; *π*_*s*_, silent sites; *π*_*a*_, nonsynonymous sites), number of haplotypes (*n*_*h*_), haplotype diversity (*H*_*e*_), and population differentiation (*F*_*ST*_) within each region and the total for the investigated populations of *Pinus krempfii*

		Longitude	Latitude		Nuclear gene								*cp*SSR					*mt*DNA
							
Populations		(^o^E)	(^o^N)	*N*^1^	*S*	*n*_*h*_	*θ*_*w*_	*θ*_*ws*_	*π*_*t*_	*π*_*s*_	*π*_*a*_	*F*_*ST*_	*n*_*h*_	*H*_e_	*G*_*ST*_	*R*_*ST*_	*F*_*ST*_	*n*_*h*_
1	Da Chay 89A	108.6843	12.1758	9	27	18	0.0009	0.0012	0.0010	0.0013	0.0004		9	1.000				1
2	Da Chay 90A	108.7015	12.1756	13	38	25	0.0011	0.0015	0.0010	0.0014	0.0005		9	0.936				1
3	Da Chay 91B	108.6893	12.1938	12	37	24	0.0011	0.0015	0.0010	0.0013	0.0004		6	0.803				1
Total Da Chay				34	52	65	0.0012	0.0017	0.0010	0.0014	0.0004	0.038*	15	0.906	0	−0.004	0.007	1
4	Cong Troi 102	108.4095	12.091	3	17	6	0.0008	0.0012	0.0009	0.0012	0.0004		3	1.000				1
5	Cong Troi 103	108.4667	11.9488	10	32	20	0.0010	0.0013	0.0011	0.0014	0.0006		8	0.956				1
Total Cong Troi				13	38	26	0.0011	0.0016	0.0011	0.0014	0.0005	0.078*	11	0.974	NA	NA	0.221*	1
6	Bidoup	108.6854	12.0475	10	35	20	0.0011	0.0016	0.0011	0.0016	0.0004		4	0.778				1
Total				57	67	111	0.0014	0.0020	0.0011	0.0015	0.0004	0.052**	22	0.911	0.016	0.017	0.062*	1

1 Number of sampled individuals. The sample size should be 2*N* for diploid nuclear genome and *N* for haploid *cp* and *mt*DNA. NA, not calculated due to small sample size.

* *P* < 0.05;

** *P* < 0.01.

### DNA extraction, amplification, and sequencing

Total DNA was extracted from needles using a Plant Genomic DNA Kit (Tiangen Biotech, Beijing, China). The search for suitable loci began with 132 nuclear genes (Gao et al. 2012) in 16 individuals of *P. krempfii*. Only the loci that were represented by a single polymerase chain reaction (PCR) band were selected. The PCR products were cloned into a pGEM–T Easy Vector (Promega, Fitchburg, WI), and 6–8 clones were sequenced for each locus to examine whether they consisted of a single sequence and investigate the level of polymorphism at these loci in the 16 sequenced individuals. Finally, ten polymorphic loci were selected for population analysis. The putative function and structure of these loci and the PCR primers are described in Table S1. PCR products were separated by agarose gel electrophoresis, and the desired products were cut from the gel and purified. The purified products were directly sequenced using an ABI 3730 automated sequencer (Applied Biosystems, Foster City, CA). When sequences had two or more heterozygous sites, the purified PCR products were first cloned into the pGEM-T Easy Vector System (Promega, Fitchburg, WI). Then, 6–8 clones were sequenced to determine individual alleles for each individual. The sequence of each allele was decided when at least two independent clones had identical sequence.

For the chloroplast genome, we first screened for polymorphisms at 13 microsatellite loci (*cp*SSR) using 16 individuals. Subsequently, seven polymorphic loci were chosen for population analysis. Primer sequences, annealing temperatures, and sizes of each product are listed in Table S2. For six *cp*SSR loci, PCR products were separated on a CEQ8000 capillary sequencer (Beckman-Coulter, Brea, CA), and haplotypes were identified by assessing the length of fragments using the CEQ8000 fragment analysis software (Beckman Coulter). For the other seven *cp*SSR loci, PCR products were purified and sequenced directly using an ABI 3730 automated sequencer (Applied Biosystems), and haplotypes were scored according to the number of repeat units.

For the mitochondrial genome, we first tested 14 mitochondrial DNA (*mt*DNA) regions using 16 individuals. Of these 14 regions, ten were successfully amplified and then sequenced for all individuals. The purified PCR products were directly sequenced using an ABI 3730 automated sequencer (Applied Biosystems). The primer sequences, annealing temperature, and sizes of each product are listed in Table S2.

### Genetic diversity, linkage disequilibrium, and neutrality tests

Sequences were aligned using the BioEdit v. 7.0.5.2 program (http://www.mbio.ncsu.edu/bioedit/page2.html). Genetic variation at nuclear loci was assessed using the DnaSP v. 5.10.01 program (Librado and Rozas 2009). The following parameters were calculated: the number of segregating sites (*S*), nucleotide polymorphism (Watterson 1975) at total sites (*θ*_*w*_), silent sites (*θ*_*ws*_), and nucleotide diversity (*π*) (Nei 1987) at total sites (*π*_*t*_), silent sites (*π*_*s*_), and nonsynonymous sites (*π*_*a*_). Measures of linkage disequilibrium (*r*^2^) among informative sites were calculated using DnaSP. The statistical significance of LD was determined by Fisher's exact tests with Bonferroni correction. The overall decay of LD with physical distance across ten loci was evaluated by nonlinear regressions of *r*^2^ on the distance between sites in base pairs (Remington et al. 2001). Inbreeding coefficient (*FIS*) (Slatkin 1991) averaged across populations was computed using Arlequin v. 3.5.1.2 program (Excoffier and Lischer 2010).

Nuclear loci were tested for departures from neutrality using Tajima's *D* (Tajima 1989), Fu and Li's D* and F* (Fu and Li 1993), Fu's *Fs* (Fu 1997), and the standardized Fay and Wu's *H* (Fay and Wu 2000; Zeng et al. 2006) statistics, as well as the McDonald and Kreitman (MK) test (McDonald and Kreitman 1991). The aforementioned statistics were calculated using DnaSP program. Orthologous sequences from *P. parviflora* were used as outgroups for the *H* and MK tests. The significance of each test was determined using 1000 coalescent simulations.

For *cp*SSR size scores, the seven *cp*SSR loci were combined to derive the haplotype for each individual. All genetic diversity analyses were based on individual haplotypes. The observed number of haplotypes and haplotype diversity (*H*_*e*_) were calculated for each population, region, and the species using the Arlequin program.

Mitochondrial DNA sequences for individual loci were aligned and then combined to generate a concatenated sequence for each individual. As no polymorphism was detected across all loci, we obtained only one concatenated mitotype. Thus, no further analysis was performed on the *mt*DNA sequences.

### Population structure

The degree of population differentiation for nuclear and *cp*SSR loci was assessed using the hierarchical analysis of molecular variance (AMOVA) (Excoffier et al. 1992). The differentiation was quantified using the *FST* statistic (Wright 1951). The statistical significance of this statistic was tested using a permutation procedure with 10,000 replications using Arlequin program.

For the nuclear genome, the population structure was further investigated using the model-based clustering algorithm implemented in the Structure v. 2.3 program (Hubisz et al. 2009). This program uses a Bayesian algorithm to infer population clustering given the number of clusters (*K*) in a sample of individuals. The most likely number of clusters (*K*) was determined using the *ΔK* method (Evanno et al. 2005), in which *ΔK* is an *ad hoc* statistic based on the rate of change in the log probability of data between successive *K* values. The chosen value of *K* was the one that gave the highest value of *ΔK*. Ten replicate runs were conducted for every value of *K* between one and ten, with a burn-in of 50,000 and a run length of 500,000 iterations. An admixture model was used without prior population information. Only sites between which Fisher's exact tests (with Bonferroni correction) showed no significant correlation were used in the Structure analysis (see *Genetic variation*, *linkage disequilibrium and neutrality tests* for details).

For the *cp*SSR data, population structure was analyzed by comparing two coefficients of population divergence (*GST* and *R*_ST_). *GST* is based on haplotype frequencies only, while *R*_ST_ takes into account similarities or relatedness among haplotypes. Thus, a significantly higher *R*_ST_ value than *G*_ST_ means that closely related haplotypes are geographically close to each other, indicating the presence of a population structure. The program Permut & CpSSR v. 2.0 (Pons and Petit 1996) was used to compare *G*_*ST*_ *vs*. *R*_*ST*_ using 10,000 random permutations. We further assessed the genetic structure using *cp*SSR data by spatial analysis of molecular variance of haplotype distribution using the SAMOVA v. 1.0 program (Dupanloup et al. 2002). This program implements a simulated annealing approach to define groups of populations (*K*) that maximize the proportion of total divergence due to differences between groups of populations (*F*_*CT*_). In this analysis, *K* = 2–5 were tested to search for the *K* that gave the highest *F*_*CT*_ or for which *F*_*CT*_ reached plateau. The significance of *F*_*CT*_ value was tested by simulating the annealing process 1000 times.

### Demographic history of *P. krempfii*

For nuclear loci, we used approximate Bayesian computation (ABC) to infer the demographic history of *P. krempfii*. Various demographic scenarios were fitted to the observed sequence data following the procedure described by Ingvarsson (2008). Briefly, a large number of replicate simulations were performed for each demographic model, where the parameters of the model were drawn from prior distributions. Simulated data were summarized using *θ*_*w*_ (Watterson 1975), Tajima's *D* (Tajima 1989), the standardized Fay and Wu's *H* (Fay and Wu 2000; Zeng et al. 2006), and Kelly's *Z*_*nS*_ (Kelly 1997) statistics. The same set of summary statistics was calculated for the observed data. The simulated samples were accepted only when they were sufficiently close to the observed data. The accepted data points were then used to estimate the posterior distribution for the parameters of the model (Beaumont et al. 2002). Model selection was conducted as described by Beaumont et al. (2002) using the VGAM package in R (http://cran.r-project.org/web/packages/VGAM/). We tested the following three demographic models: (1) standard neutral model; (2) exponential growth model; and (3) bottleneck model. The standard neutral model assumes stable population size and contains only two parameters: *θ* and *ρ*. The exponential growth model assumes an increase of the ancestral population of size *N*_*1*_ exponential to the current population size (*N*_0_) starting at time *T*_0_ with a constant exponent (*α* = log (*N*_0_/*N*_1_)/*T*_0_). In the bottleneck model, the ancestral population size is assumed to be same as that of the current population (*N*_0_) and then shrinks due to a bottleneck with a subsequent exponential expansion. The bottleneck was characterized by three parameters: the time since the end of the bottleneck (*T*_0_), the duration of the bottleneck (*T*_*d*_), and the reduction in population size during the bottleneck (*N*_1_). The growth rate (*α*) after the bottleneck was given by the function *α* = log (*N*_0_/*N*_1_)/*T*_0_. The prior ranges of *N*_1_, *T*_0,_ and *T*_*d*_ were chosen to cover a broad range of possible demographic scenarios (Table S3). In all simulations, locus-specific *θ* and *ρ* values were used. They were derived by multiplying the length of each gene (*L*) by the *per site* values of *θ* and *ρ*, respectively. The values of *θ* and *ρ per site* were drawn from uniform priors covering ranges of 10^−5^−0.05 and 10^−5^−0.1, respectively (Table S3). For model selection, 3 × 10^5^ simulations were run for each of the three demographic models and the 900 points closest (*P*_*δ*_ = 0.001) to the obtained data were used. An additional 7 × 10^5^ samples were subsequently simulated for the growth model. In total, 10^6^ samples were generated for the growth model and 1000 closest data points (*P*_*δ*_ = 0.001) were used to estimate the posterior distributions of the model parameters. We tested different values of *P*_*δ*_ (0.01–0.0005) but obtained similar posterior modes for the estimated parameters (data not shown), confirming that the ABC estimates were insensitive to *P*_*δ*_ (Beaumont et al. 2002). Finally, we used posterior predictive simulations (Gelman et al. 2004) to assess the fit of the parameters estimated from the posterior distributions. Using parameters sampled from the posterior distributions, 100,000 sets of new data were generated. These simulated data sets were summarized using *θ*_*w*_, Tajima's *D*, the standardized Fay and Wu's *H* (using corresponding sequences of *P. parviflora* as an outgroup), and Kelly's *Z*_*nS*_ and then compared to the corresponding observed data. All simulations were performed and analyzed using the *ms* program (Hudson 2002). The ABC analyses were performed using R scripts provided by Beaumont (http://www.rubic.rdg.ac.uk/∼mab/stuff/).

The historical population expansion events for *cp*SSR data were tested using mismatch distribution analysis using Arlequin program. *cp*SSR data were coded in a binary fashion following Navascues et al. (2006). A total of 10,000 parametric bootstrap replicates were used to generate an expected distribution under a model of sudden demographic expansion and to test the goodness of fit of the demographic model. The sum of squared deviations (SSD) between the observed and expected mismatch distributions was computed, and *P*-values were calculated from the proportion of simulations producing an SSD value that was greater than the experimental value. The raggedness index and its significance were also calculated to quantify the smoothness of the observed mismatch distribution.

Isolation by distance between populations for nuclear and *cp*SSR loci was tested by regressing pairwise population differentiation *F*_*ST*_ against the geographic distance between populations (Mantel test), with 10,000 random permutations using Arlequin program.

## Results

### Genetic variation, linkage disequilibrium, and neutrality tests

Ten nuclear loci were sequenced for 57 individuals of *P. krempfii*. The size of the sequenced fragments ranged from 494 to 1252 bp with a total concatenated length of 8950 bp (Table S1). The lengths of coding (exon) and noncoding (intron) regions were 4608 bp and 4342 bp, respectively. The levels of polymorphism varied about 10 to 46-fold among loci. The *GSTH*2 locus was the most polymorphic (*θ*_*w*_ = 0.0030, *π*_*t*_ = 0.0046), while the *TPP*1 locus the least polymorphic (*θ*_*w*_ = 0.0003, *π*_*t*_ = 0.0001) (Table S4). Averaged across all loci, *P. krempfii* exhibited an extremely low level of nucleotide polymorphism (*θ*_*w*_ = 0.0014, *π*_*t*_ = 0.0011). The nucleotide diversity at silent sites (*π*_*s*_ = 0.0015) was approximately four times of that at nonsynonymous sites (*π*_*a*_ = 0.0004). Low level of LD was observed across the ten investigated nuclear loci with an average *r*^2^ = 0.1. LD decayed fast, with *r*^2^ dropping below 0.1 within about 100 bp (Fig. S1).

With only a few exceptions at the species level, neutrality tests yielded nonsignificant values of Tajima's *D*, Fu and Li's D* and F*, Fu's *Fs*, and Fay and Wu's *H* (Table S5). MK tests could not be performed for most of the loci due to low polymorphism and yielded nonsignificant results for others. In brief, we did not find evidence for deviations from neutrality at the analyzed nuclear loci.

For the seven *cp*SSR loci, we sequenced all SSR size variants to confirm that they were caused by variation in the number of repeat units. This was indeed the case, except for one point mutation detected in the SSR area of the Pt100783 locus, and two point mutations between PKS108222A and PKS108222T loci. Due to potentially different model of evolution between *cp*SSR and point mutations, these point mutations were not considered in the analysis. Three to four haplotypes were detected at each *cp*SSR locus. When all loci were combined, they defined 22 haplotypes, of which 11 were found only once. Haplotype diversity was high at both population (*H*_*e*_ = 0.778–1.000) and species levels (*H*_*e*_ = 0.911, Table 1). For *mt*DNA, all 57 individuals were monomorphic across all 10 regions (approximately 10 kbp) (Table 1).

### Genetic differentiation and population structure

Population differentiation (*F*_*ST*_) was low across most nuclear loci. Significant *F*_*ST*_ values were detected for only four loci: *CFX*, *SOS*27, *GSTG*1, and *GSTH*2 (Table S6). High *F*_*ST*_ value for the *SOS27* locus (0.204) was mainly caused by the population Cong Troi 102. Only three individuals were sampled for this population, and two of them shared the same haplotype that was distinct from those of all other individuals. After removing Cong Troi 102 population, the *F*_*ST*_ was reduced to 0.018. The multilocus *F*_*ST*_ value for *P. krempfii* was significant but low (*F*_*ST*_ = 0.052). Structure analysis failed to reveal any meaningful grouping pattern. First, the highest log probability of data *L(K)* was detected at *K* = 1. Second, although the maximum of *ΔK* was found at *K* = 5, each population had fairly admixed ancestry from all five genetic clusters (Fig. S2).

Similar to nuclear loci, population divergence based on the *cp*SSR data was very low (*G*_*ST*_ = 0.016, *F*_*ST*_ = 0.06) (Table 1). Comparisons of *G*_*ST*_ versus *R*_*ST*_ indicated that *R*_*ST*_ was not significantly greater than *G*_*ST*_, which rejected the presence of a phylogeographic structure in the investigated populations. Correspondingly, SAMOVA analysis failed to reveal any meaningful phylogeographic grouping; *F*_*CT*_ increased steadily with *K* value from two to five, and no inflexion was detected (data not shown). For both nuclear and *cp*SSR loci, Mantel test revealed no correlation between the pairwise genetic distances (*F*_*ST*_) and geographic distances (*P* > 0.05).

### Demographic history of *P. krempfii* populations

The ABC model selection approach suggested population expansion in *P. krempfii*. The posterior probability for the growth model (0.776) was higher than those for standard neutral and bottleneck models (0.002 and 0.222, respectively). Parameters of the growth model had distinct modes in the posterior distributions (Table 2). Posterior predictive simulations showed a generally good agreement between the observed and simulated data sets (Fig. S3). The posterior mode of ancestral population size (*N*_1_, in units of *N*_0_) was 0.0318 (95% credible interval 0.0118–0.2086), and the time of initial size change (*T*_0_, in units of 4*N*_0_ generations) was 0.8593. Using all silent polymorphic sites (*θ*_*ws*_ = 0.002) and mutation rate per generation (*μ*), we directly calculated current population size (*N*_e_) of *P. krempfii* as *θ*_*ws*_/4*μ*. Assuming generation time of 50 years and mutation rate per year of 7 × 10^−10^ estimated for the genus *Pinus* by Willyard et al. (2007), the estimated population size for *P. krempfii* (1.43 × 10^4^) was very small. Based on this estimated population size and a generation time of 50 years, the *T*_0_ would correspond to 2458 years with a 95% credible interval of 117–65008 years (Table 2). The *cp*SSR mismatch distribution test also indicated that a recent expansion model could be accepted for *P. krempfii* (*P*_(SSD)_ = 0.526).

**Table 2:** Posterior distributions for the demographic parameters of the exponential growth model estimated by ABC analysis based on ten nuclear loci

Parameter	Mode	2.5%	97.5%
*θ*	0.0299	0.0045	0.0534
*ρ*	0.0346	0.0008	0.0997
*N*_1_	0.0318	0.0118	0.2086
*T*_0_	0.8593 (2458)	0.0408 (117)	22.730 (65,008)

Modes 2.5% and 97.5% are medians of the lower and upper bounds of the estimated 95% posterior density credibility interval, respectively; *θ,* per site nucleotide polymorphism; *ρ,* per site recombination rate; *N*_*1,*_ ancestral population size (in units of current population size *N*_0_); *T*_0,_ the time of the initial size change; *T*_0,_ is in units of 4*N*_0_ generations and scaled by 10^−3^; values in parentheses are converted *T*_0_ in years assuming *N*_0_ of 1.43 × 10^4^ for *P. krempfii* and generation time of 50 years.

## Discussion

### Genetic diversity and population demography in *P. krempfii*

**Figure 3:**
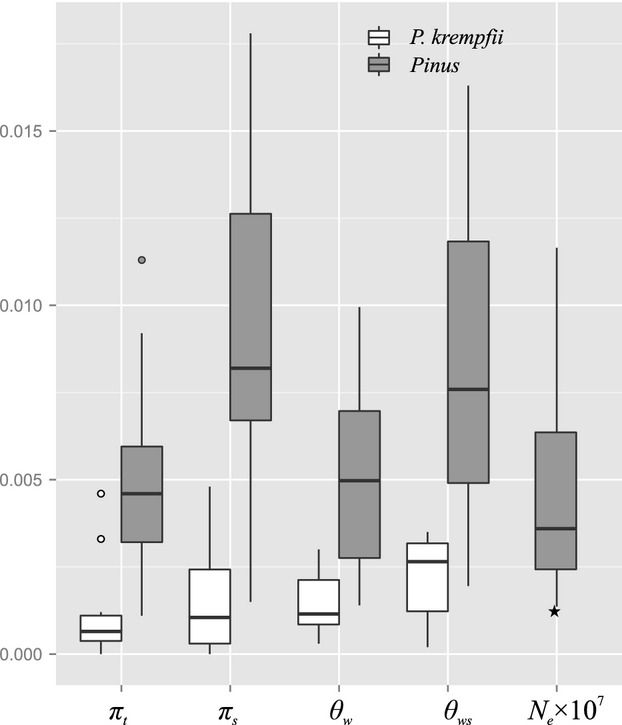
The distribution of nucleotide diversity and effective population size (*N*_*e*_) for *Pinus krempfii* and genus *Pinus* based on nuclear genes. *π*_*t,*_ nucleotide diversity measured at all sites; *π*_*s*_, nucleotide diversity at silent sites; *θ*_*w,*_ nucleotide polymorphism at all sites; *θ*_*ws*_, nucleotide polymorphism at silent sites. *N*_*e*_ (effective population size) was calculated based on *θ*_*ws*_/4*μ* assuming generation time of 50 years and mutation rate per year of 7 × 10^−10^ estimated for the genus *Pinus* by Willyard et al. (2007). The value of *N*_*e*_ for *P. krempfii* was denoted by star. For species where multiple reported data were available, we used the average (see Table S7 for details).

Our analysis revealed extremely low levels of nucleotide polymorphism in *P. krempfii*. For nuclear loci, the mean silent nucleotide diversity in *P. krempfii* (*π*_*s*_ = 0.0015; *θ*_*ws*_ = 0.0020) was comparable with those found in *Pinus cembra* (*π*_*s*_ = 0.0024; *θ*_*ws*_ = 0.0024) (Mosca et al. 2012), but much lower than those in other pines (Fig. 3; Table S7). For *mt*DNA, we did not detect any polymorphism across ten *mt*DNA regions (approximately 10 kbp). Although low nucleotide variation for *mt*DNA has been observed in conifers, some of the *mt*DNA regions analyzed in this study have been widely used in previous population studies in pines, and various levels of polymorphism have been reported for most pine species, including those with limited range of distribution (Chiang et al. 2006; Eckert et al. 2008; Wang et al. 2011). For example, Eckert et al. (2008) detected 14 mitotypes in *Pinus balfouriana*, a California endemic pine with only two disjunct populations, based on four *mt*DNA fragments involved in this study. For the *cp*SSR, haplotype diversity (*H*_*e*_ = 0.911) detected in *P. krempfii* was high, as observed in most pine species (Höhn et al. 2005; Petit et al. 2005; Wang et al. 2011, 2013). The contrasting levels of genetic diversity between *cp*SSR and *mt*- and nuclear DNA sequences observed in *P. krempfii* can be due to the different mutation rates between genomic regions. In pine species, the mutation rate for length variation at *cp*SSR loci (3.2–7.9 × 10^−5^) was 5–6 orders of magnitude higher than the substitution rates in *mt*- (4 × 10^−11^) and nuclear DNA (7 × 10^−10^) sequences (Provan et al. 1999; Mower et al. 2007; Willyard et al. 2007). The asymmetric diversity between genetic markers has been observed in other pines such as *P. cembra* using *cp*SSR (*H*_*e*_ = 0.917) and nuclear DNA sequences (*π*_*s*_ = 0.0024) (Höhn et al. 2005; Mosca et al. 2012). The difference in diversity between *cp*SSR relative to *mt* and nuclear loci could also be due to varied demographic and selective histories of different genomes. For example, during range fragmentation, the loss of the *cp*DNA diversity in single spatially isolated population could be compensated by efficient pollen flow from adjacent populations, whereas isolated populations may experience stronger bottleneck on *mt* genome due to limited seed dispersal. Natural selection could reduce the genetic diversity of functional nuclear loci, but may not affect neutral *cp*SSR loci. In summary, the nucleotide polymorphism in nuclear and *mt* genomes of *P. krempfii* was lowest among the pine species studied so far, whereas high genetic diversity was observed at *cp*SSR loci possibly due to the hypervariable nature of the SSR markers.

We detected low (5.2%) but significant differentiation among the extant populations of *P. krempfii*. Even within each region, *F*_*ST*_ values (3.8–7.8%) were also significant. This level of differentiation is comparable to pine species with wide distribution ranges (Wang et al. 1991; Ma et al. 2006; Pyhäjärvi et al. 2007). Due to lack of geographical barrier between sampled populations of *P. krempfii*, the population differentiation in this species could have been caused by fragmented nature of its distribution. Unlike most other pines, *P. krempfii* does not form pure stands, and individual populations consist of small groups of trees and/or solitary individuals dispersed among dense thicket of other tree species (Nguyen and Thomas 2004). These conditions are likely to limit dispersal of its pollen and seed, and contribute to differentiation between local populations. Furthermore, *P. krempfii* is distributed in a wet rainforest environment, which could preclude efficient wind pollination (Turner 2001). High humidity dampens pollen grains, and heavy rains wash away pollen from the air. In summary, despite relative proximity of individual populations, the low population density of *P. krempfii* and humid environment have prevented gene flow and led to certain degree of population differentiation. These findings indicate that even species with a very limited distribution may harbor genetically differentiated populations.

Approximate Bayesian computation simulations using nuclear loci suggested that *P. krempfii* experienced an exponential population growth, which started approximately 2450 years ago. Population expansion was also supported by mismatch distribution test based on the *cp*DNA data. The timing of the population growth in *P. krempfii* was much later than those in other Eurasian pines, which were dated at a few hundred thousand years ago, for example, *P. densata* from the Tibetan Plateau (Gao et al. 2012) and *P. sylvestris* from Europe (Pyhäjärvi et al. 2007). Thus, the population expansion revealed in *P. krempfii* might have been induced by regional climate changes or human activates, rather than global climate fluctuations during the Pleistocene. Assuming generation time of *P. krempfii* as 50 years, the population growth lasted for only 49 generations in this species. This episode of population expansion is too short to allow for accumulation of extensive polymorphism. Moreover, the habitat of *P. krempfii* has deteriorated and become fragmented in the last decades (Nguyen and Thomas 2004), which could have resulted in the reduction and fragmentation of *P. krempfii* populations. As suggested by earlier studies, the models implemented and explored in ABC and mismatch distribution analyses are most likely too simplistic (Ingvarsson 2008; Gao et al. 2012). Presumably, *P. krempfii* has gone through repeated population size expansions and contractions, and the most recent population decline was not revealed by the current simulations. The reduction in population size and population fragmentation could decrease the frequency of rare alleles in a very short time (Ellstrand and Elam 1993).

The extremely low nucleotide diversity detected in *P. krempfii* is nearly 2–8 times lower than in most other Eurasian pines (Fig. 3; Table S7) and is consistent with its small population size (1.43 × 10^4^). ABC analyses suggested that *P. krempfii* has maintained an extremely small ancestral population, comprising only a few hundred individuals (455) for more than 2.8 Myr before entering the population growth phase. This situation of *P. krempfii* with small ancestral population size is different from that of other relic gymnosperms such as *Ginkgo biloba* and *Cathaya argyrophylla*, which have been abundant and widespread before glaciations (Wang and Ge 2006; Gong et al. 2008). Brodribb and Feild (2008) speculated that competition from angiosperms and subtropical podocarps could have limited the success of *P. krempfii*.

Small population size has two important genetic consequences. One is loss of genetic diversity due to genetic drift. Another is increased inbreeding, which leads to higher levels of homozygosity and mortality caused by lethal or semi-lethal alleles. The inbreeding coefficient (*FIS* = 0.26) in *P. krempfii* is much higher than those in other pines such as *P. pinaster* (0.069) (Eveno et al. 2008). In this study, we collected cones from most of the sampled individuals and found that practically all of the seeds were empty. Although the mating system of *P. krempfii* has not been studied, pine species are self-compatible and it is well known that the presence of empty seed in this group of conifers is a sure indicator of increased levels of inbreeding (Karkkainen et al. 1996). Therefore, apart from the loss of diversity due to genetic drift, the extant populations of *P. krempfii* may be also suffering from additional loss due to inbreeding. Future study on the mating system of *P. krempfii* could reveal the true impact of inbreeding on the loss of genetic diversity in this species.

*Pinus krempfii* is thought to be an ancient relict (Nguyen and Thomas 2004). It is the only extant species in subsection *Krempfianae* and diverged from other pines more than 10 million years ago (Willyard et al. 2007). The unique morphology, physiology, anatomy, limited distribution range, and distinct habitat also indicate that this species has been isolated from the other pines for a long time. Long-term isolation together with small population size could have enhanced the impact of genetic drift and inbreeding in *P. krempfii*, resulting in severe reduction in genetic diversity.

Nucleotide diversity could also be reduced by selective sweeps that diminish variation at and around particular genes or by purifying selection against deleterious mutations closely linked to neutral variants (Hahn 2008). However, we did not find strong evidence for selection at any of the analyzed loci. The rapid decrease of LD over distance also suggested limited effects of genetic hitchhiking. Therefore, while selection may partly explain the low levels of nucleotide variation at several loci, it does not seem to be sufficient to explain the low levels of variation across nuclear loci included in our study.

### Conservation implications

The low nucleotide polymorphism, restricted distribution, and high ratio of empty seeds in *P. krempfii* suggest the species is exposed to a considerable risk of extinction. Although the most extant populations of *P. krempfii* are currently under legal protection in national parks in Vietnam, they face serious threat and risk of extinction by stochastic processes because of their small size. Population size is the most important of the five criteria for listing species as endangered under the International Union for the Conservation of Nature and Natural Resources (IUCN) system (http://www.iucn.org/), and the loss of genetic variation may decrease the potential for a species to persist in the face of biotic and abiotic changes. Thus, efforts should be made to increase the genetic diversity and population size of *P. krempfii*. *Pinus krempfii* does not form pure forests and typically occurs as small groups of 10–30 trees in dense subtropical forests (Nguyen and Thomas 2004). The persistence and regeneration of this species are highly reliant on the subtropical forest environment. For example, the seedlings and saplings of *P. krempfii* were restricted to shade environment under the forest canopy (Nguyen and Thomas 2004). Unfortunately, there is a continuing decline in the extent and quality of its habitats due to human activities (e.g., war in the 1960s and the clearance of land for agriculture) and climate changes in recent decades (Nguyen and Thomas 2004). The loss of habitat could have decreased the population size of *P. krempfii* in the past and would prevent recovery of population in the future. Therefore, the first effort to recover the extant *P. krempfii* population should be the protection and restoration of the habitat that *P. krempfii* is adapted to.

The *in situ* conservation alone, however, cannot conserve and recover the species because of the restricted distribution of *P. krempfii*. Therefore, *ex situ* conservation should also be given high priority to offset the habitat deterioration and fragmentation. In this regard, introductions can be designed to establish self-sustaining wild populations, and this practice should be carried out in suitable habitats.

The high ratio of empty seed detected in *P. krempfii* suggests that this species is suffering from inbreeding depression. Thus, traditional breeding practices such as controlled crosses between genetically distinct populations, even between individuals of the same stand, could be helpful to restore and enrich genetic diversity in *P. krempfii*. Controlled crosses are important genetic tools for both breeding and conservation of wild populations of economically and ecologically important plant species. Although population differentiation was low in *P. krempfii*, some population pairs (e.g., Bidoup *vs*. Cong Troi 103) showed considerable divergence (data not shown). Therefore, controlled crosses between these populations seem reasonable, and a seed orchard could be established for production of genetically improved seeds of *P. krempfii*, but the potential benefits should be evaluated prior to full implementation. Future studies should employ both genomic and ecological data to better understand the evolutionary history of *P. krempfii* and to make additional conservation efforts (e.g., outcrossing assessment and population viability analysis) to develop better quantitative recovery criteria for this species.

## Data Archiving

DNA sequence: GenBank accessions KJ711909-KJ713057.

Frequency of each mitotype and chlorotype in all six population: Dryad entry doi:10.5061/dryad.dr283.

## Supporting Information

**Table S1.** Descriptions of the ten investigated nuclear loci. **Table S2.** Primers used for *mt*DNA and *cp*DNA amplification. Primers that generated polymorphic *cp*SSR sites are in bold. **Table S3.** Prior distribution of the demographic parameters for standard neutral model (N), exponential growth model (G), and bottleneck model (B). **Table S4.** Geographic location, sample sizes (*N*), the number of segregating sites (*S*), nucleotide polymorphism (*θ*_*w*_), nucleotide diversity (*π*_*t*_, total sites; *π*_*s*_, silent sites; *π*_*a*_, nonsynonymous), number of haplotypes (*n*_*h*_) of the investigated populations of *P. krempfii* based on individual nuclear loci. **Table S5.** Neutrality tests for individual nuclear loci as measured by Tajima's *D*, Fu and Li's *D** and *F**, Fu's *Fs*, standardized Fay and Wu's *H*, and the MK test. **Table S6.** Population differentiation (*F*_*ST*_) for each region and total sample of *P. krempfii* based on individual and combined nuclear loci. **Table S7.** Summary of nucleotide diversity and effective population size in 22 pine species based on nuclear genes. Species with limited range of distribution are in bold. **Figure S1.** Linkage disequilibrium (*r*^2^) as a function of the distance between sites across the ten nuclear genes in *P. krempfii*. **Figure S2.** Structure analysis of the six sampled populations of *P. krempfii* based on nuclear loci. (a) The log probability of data *L(K)* (mean and standard deviation over 10 replicates) given the number of genetic clusters *K* ranging between 1 and 10. (b) Magnitude of Δ*K* for each *K* = 2–9. (c) Assignments of population frequency of demes by structure at *K* = 2–6. For each *K* value, results of the run with the highest value of *L(K)* were used. **Figure S3.** Mean and variance of four summary statistics calculated from 10^5^ posterior predictive simulations. Values of the corresponding summary statistics for the observed nuclear data are indicated by vertical red lines.
