# Supplementary material for: Extremely low nucleotide polymorphism in Pinus krempfii Lecomte, a unique flat needle pine endemic to Vietnam
Source: Ecol Evol. 2014 May 8;4(11):2228–38. doi: 10.1002/ece3.1091 (PMC4201436; doi:10.1002/ece3.1091)
Supplement: Supplementary file 1 [file ece30004-2228-sd1.pdf]

Supporting information

Table S1 Descriptions of the ten investigated nuclear loci**.**

Table S2 Primers used for *mt*DNA and *cp*DNA amplification. Primers that generated polymorphic *cp*SSR sites are in bold.

Table S3 Prior distribution of the demographic parameters for standard neutral model (N), exponential growth model (G) and bottleneck model (B).

Table S4 Geographic location, sample sizes (*N*), the number of segregating sites (*S*), nucleotide polymorphism (θ_w) nucleotide diversity (π_t, total sites; π_s, silent sites; π_a, non-synonymous), number of haplotypes (n_h) of the investigated populations of *P. krempfii* based on individual nuclear loci.

Table S5 Neutrality tests for individual nuclear loci as measured by Tajima's *D*, Fu and Li's *D** and *F**, Fu's *Fs*, standardized Fay and Wu's *H*, and the MK test.

Table S6 Population differentiation (F_{ST}) for each region and total sample of *P. krempfii* based on individual and combined nuclear loci.

Table S7 Summary of nucleotide diversity and effective population size in 22 pine species based on nuclear genes. Species with limited range of distribution are in bold.

Fig. S1 Linkage disequilibrium (r^2) as a function of the distance between sites across the ten nuclear genes in *P. krempfii*.

Fig. S2 Structure analysis of the six sampled populations of *P. krempfii* based on nuclear loci. (a) The Log probability of data *L(K)* (mean and standard deviation over 10 replicates) given the number of genetic clusters *K* ranging between 1 and 10. (b) Magnitude of ΔK for each *K* = 2-9. (c) Assignments of population frequency of demes by Structure at *K* = 2-6. For each *K* value, results of the run with the highest value of *L(K)* were used.

Fig. S3 Mean and variance of four summary statistics calculated from 10^5 posterior predictive simulations. Values of the corresponding summary statistics for the observed nuclear data are indicated by vertical red lines.

Table S1 Descriptions of the ten investigated nuclear loci.

Locus	Putative function	PCR primers (5'-3')	Annealing temperature (^oC)	Alignement length* (bp)	Exon	Intron
IFG 8612	Late embryogenesis abundant protein	F: GAGTGCAAATAGCTACTCTCAGG	52	966	811-966	1-810
		R: CAACAATAGGAATGTGCATGGTAAGG				
		IF: CAACTGCCCTGTCAATAGGTC				
		IR: GACCTATTGACAGGGCAGTTG				
CFX	Cellulose synthase	F: ATCCAAGGGCCAGTGTATGT	52	1102	1-203; 1067-1102	204-1066
		R: CAAACGACTTCTCAAAGCTTCTCT				
		IF: TATTGAGGAGGATATACCTG				
		IR: GTAAGAGCTTCAACTACAGA				
4*CL*	4-coumarate-CoA ligase	F: CAGAGGTGGAACTGATTTC	52	1089	1-808; 1085-1089	809-1084
		R: CTGCTTCTGTCATGCCGTA				
		IF: CATCTATTCTCTCAATTCCG				
		IR: TTTGTGATGTCCAGGACAAT				
TPP 1	Sucrose-phosphatase	F: AAAGAGCTGAGAAAGGAGAAAC	60	1195	1-161; 369-470; 784-978	162-368; 471-783; 979-1195
		R: CCTCATACCACTGCAATAACTC				
		IF: CTATACCTGAATTGGTACAG				
		IR: GTCATGCAAGGAACTTAATC				
IFG 1934	Chloroplast light harvesting chlorophyll a/b binding protein	F: CTACTGCTGCAACAATGGCAA	52	765	1-765	-
		R: TAGGCCCAGGCGTTGTTGTT				
SOS 27	Calcium-dependent protein kinase	F: AGAAACGTCTTAAGGCTGCTG	60	1252	1-9; 146-261; 337-513	10-145; 262-336; 514-1252
		R: TGGTTGAAATTGTGATCCCTT				
Rav 2	Ethylene-responsive element binding factor	F: GTTGCAGGAATTGATTGTGTG	60	752	38-661	1-37; 662-752
		R: AATGGCAGAGACCAAGTTCTG				
GSTG 1	Gamma subunit of elongation factor 1b subfamily	F: GCAACCAAGGAACAAAAGC	55	815	1-204; 471-815	205-470
		R: TCGAATCTAACAAAGGCTCAC				
GSTH 2	DHAR class glutathione S-transferase	F: ATGTCAGACCTCGAAGTTTTTG	55	494	1-48; 163-242; 441-494	49-162; 243-440
		R: ATTTTATCAGAGTCTTGGATCCAT				
TPS 4	Alpha-trehalose-phosphate synthase	F: CATGGGTCAATTAGAATCTGTG	55	520	1-520	
		R: GATTTACCTTGATAGCACCACTTA				
Total				8950	4608	4342

* without outgroup

IF and IR, internal primers for sequencing

Table S2 Primers used for *mt* DNA and *cp* DNA amplification. Primers that generated polymorphic *cp* SSR sites are in bold.

Locus	Primer name	Sequence (5´-3´)	Annealing temperature (^oC)	Sequence length (bp)	Reference
mtDNA					
18*S rRNA*	*rrn* 18-F	GGGGTGCTTTCTTGACCATTTC	57	964	This study
	rrn 18-R	CCCTACGGCTACCTTGTTACGACT			
matR	*matR* -F	GACCGACATCGACTCATCTCAA	57	1128	This study
	matR -R	CATAATACGCCCACGCTCACT			
nad 3-*rps* 12	*nad3/rps* 12-F	GTTCGCTAGTTTGTTTGATCCC	54	673	(Soranzo *et al.*, 1999)
	nad3/rps 12-R	TCCCAGCAAATCCTTGACTC			
nad 1 intron 2	nad1-2F	CGCCCGTTTCCATTTCGTG	57	1510	This study
	nad1-2R	GTGGCTCGTCCGTGCTTTG			
nad 5 intron 4	nad5-4F	GGGGAGGTTTACAGGAGAT	54	574	This study
	nad5-4R	AGTAGTGCCAGCAGGAATG			
cox 1	cox1-F	TGGGAATCATCAACCTCA	55	1170	This study
	cox1-R	GACCACGAAGAAACGAAA			
mh 09	mh09-F	TCATCCATCCTCCAGCAACA	55	189	(Jeandroz *et al.*, 2002)
	mh09-R	TCATCCCCAGAAAGAGACAG			
mh 05	mh05-F	GGGAGTCAGCGAAAGAAGTAA	52	Failed amplification	(Jeandroz *et al.*, 2002)
	mh05-R	AGTCTCAGAGCCAGAAGCAG			
mh 09´	mh09´-F	CCATCCAGCCATGTCTCATC	52	Failed amplification	(Jeandroz *et al.*, 2002)
	mh09´-R	AGGGCTTCACATAGAGCATC			
mh 33	mh33-F	TTCCCCAGACAGAACAGATAG	52	Failed amplification	(Jeandroz *et al.*, 2002)
	mh33-R	GCTCTTAAGTGCTGGTTGATG			
mh 35	mh35-F	CGATGACATCTCTTAGCTTCC	52	Failed amplification	(Jeandroz *et al.*, 2002)
	mh35-R	TGGGGAATAGGATTCGGGTAA			
nad 5 intron 1	nad5-1F	GGAAATGTTTGATGCTTCTTGGG	55	1227	(Wang *et al.*, 2000)
	nad5-1R	CTGATCCAAAATCACCTACTCG			
nad 4 intron 3	nad4-3F	GGAGCTTTCCAAAGAAATAG	55	1939	(Dumolin-Lapegue *et al.*, 1997)
	nad4-3R	GCCATGTTGCACTAAGTTAC			
nad 7 intron 1	nad7-1F	GGAACCGCATATTGGATCAC	55	1400	(Tian *et al.*, 2010)
	nad7-1R	GTTGTACCGTAAACCTGCTC			
Total mtDNA				10774	

Table S2 continued

Locus	Primer name	Sequence (5´-3´)	Annealing temperature (ºC)	Sequence Length (bp)	Reference
cpDNA					
Scored by genotyping					
**Pt45002**	PT45002-F	AAGTTGGATTTTACCCAGGTG	52	ca. 184	(Vendramin *et al.*, 1996)
	PT45002-R2	AACCCCAAGAACAAGAGGAT			
**Pt71936**	PT71936-F	TTCATTGGAAATACACTAGCCC	52	ca. 139	(Vendramin *et al.*, 1996)
	PT71936-R	AAAACCGTACATGAGATTCCC			
Pt87268	PT87268-F	GCCAGGGAAAATCGTAGG	52	ca. 147	(Vendramin *et al.*, 1996)
	PT87268-R	AGACGATTAGACATCCAACCC			
**Pt30204**	PT30204-F	TCATAGCGGAAGATCCTCTTT	52	ca. 152	(Vendramin *et al.*, 1996)
	PT30204-R	CGGATTGATCCTAACCATACC			
PCP1289	PCP1289-F	TCCTGGTTCCAGAAATGGAG	52	Failed amplification	(Provan *et al.*, 1998)
	PCP1289-R	TAATTTGGTTCCAGAATTGCG			
PCP41131	PCP41131-F	AAAGCATTTCCAGTTGGGG	52	113	(Provan *et al.*, 1998)
	PCP41131-R	GGTCAGGATTCATGTTCTTCC			
Scored by sequencing					
Pt15169	PTS15169-F	CCCCTTGTAAAGATGAAC	50	637	This study
	PTS15169-R	AATGGCTTGGTGGTATGT			
**Pt63718**	PTS63718-F	GATCTGGATATTTCCTCCGATAC	50	ca. 806	This study
	PTS63718-R	AAGCGTTGGTTGGGTAAGC			
**Pt100783**	PTS100783-F	AAAACAATAGCGTCCTCC	50	ca. 1017	This study
	PTS100783-R	CCGAATATGGTCCAATCA			
Pt26081	PTS26081-F	ATTCAGGGATTGTAAACG	50	780	This study
	PTS26081-R	CGAGATGATAAGGGAACTA			
Pt36480	PTS36480-F	TGAAAGGGAGTAAGATAAATGG	50	833	This study
	PTS36480-R	GAGATAGATCGCAGGGAGG			
**PKS108222A***	PKS108222-F	ACCCTGAGATCGTTTGA	50	ca. 664	This study
	PKS108222-R	TCCATTGCTATGCGTTT			
**PKS108222T***	PKS108222-F	ACCCTGAGATCGTTTGA	50	ca. 664	This study
	PKS108222-R	TCCATTGCTATGCGTTT			

*PKS108222A and PKS108222T are amplified by same primers

References: Dumolin-Lapegue, S., Pemonge, M.H. & Petit, R.J. 1997. An enlarged set of consensus primers for the study of organelle DNA in plants. *Mol. Ecol.* **6:** 393-397.

Jeandroz, S., Bastien, D., Chandelier, A., Du Jardin, P. & Favre, J.M. 2002. A set of primers for amplification of mitochondrial DNA in *Picea abies* and other conifer species. *Mol. Ecol. Notes* **2:** 389-392.

Provan, J., et al. 1998. Gene-pool variation in Caledonian and European Scots pine (*Pinus sylvestris* L.) revealed by chloroplast simple-sequence repeats. *Proc. Roy. Soc. Lond. B..Biol. Sci.* **265:** 1697-1705.

Soranzo, N., Provan, J. & Powell, W. 1999. An example of microsatellite length variation in the mitochondrial genome of conifers. *Genome* **42:** 158-161.

Tian, S., Lopez-Pujol, J., Wang, H.W., Ge, S. & Zhang, Z.Y. 2010. Molecular evidence for glacial expansion and interglacial retreat during Quaternary climatic changes in a montane temperate pine (Pinus kwangtungensis Chun ex Tsiang) in southern China. *Plant Syst. Evol.* **284:** 219-229.

Wang, X.Q., Tank, D.C. & Sang, T. 2000. Phylogeny and divergence times in Pinaceae: Evidence from three genomes. *Mol. Biol. Evol.* **17:** 773-781.

Vendramin, G.G., Lelli, L., Rossi, P. & Morgante, M. 1996. A set of primers for the amplification of 20 chloroplast microsatellites in Pinaceae. *Mol. Ecol.* **5:** 595-598.

Table S3 Prior distribution of the demographic parameters for standard neutral model (N), exponential growth model (G) and bottleneck model (B).

Model	θ	ρ	N_1	T_0	T_d
N	10^{-5} - 0.05	10^{-5} - 0.1	na	na	na
G	10^{-5} - 0.05	10^{-5} - 0.1	10^{-3} - 1	10^{-5} - 1	na
B	10^{-5} - 0.05	10^{-5} - 0.1	10^{-3} - 1	10^{-5} - 1	10^{-4} - 10^{-1}

θ, *per site* nucleotide polymorphism; ρ, *per site* recombination rate; N_1, ancestral population size in exponential growth model or population size during bottleneck in bottleneck model; T_0, the time of the initial size change in growth model or the time since the end of bottleneck in bottleneck model; T_d, duration of bottleneck.

N_1 are measured in units of current population size (N_0); T_0 and T_d are measured in units of $4N_0$ generation.

θ, ρ, N_A, N_1, T_0 and T_d are given by an uniform with minimum and maximum, e.g. for all simulations, the distribution of θ is an uniform range from 10^{-5} to 0.05.

na, no paremeter in such model.

Table S4 Geographic location, sample sizes (N), the number of segregating sites (S), nucleotide polymorphism (θ_w, total sites; θ_{ws}, silent sites), nucleotide diversity (π_t, total sites; π_s, silent sites; π_a, non-synonymous), number of haplotypes (n_h) of the investigated populations of *P. krempfii* based on individual nuclear loci.

				4CL							*CFX*						
Population	Longitude (°E)	Latitude (°N)	N	S	n_h	θ_w	θ_{ws}	π_t	π_s	π_a	S	n_h	θ_w	θ_{ws}	π_t	π_s	π_a
1 Da Chay 89A	108.6843	12.1758	9	0	1	0	0	0	0	0	9	6	0.0024	0.0029	0.0030	0.0036	0
2 Da Chay 90A	108.7015	12.1756	13	5	4	0.0012	0.0022	0.0005	0.0009	0.0001	13	11	0.0031	0.0037	0.0037	0.0045	0
3 Da Chay 91B	108.6893	12.1938	12	4	5	0.0010	0.0017	0.0005	0.0009	0.0001	9	7	0.0022	0.0026	0.0025	0.0030	0
Total Da Chay			34	6	6	0.0012	0.0017	0.0003	0.0007	0.0001	13	14	0.0025	0.0030	0.0033	0.0039	0
4 Cong Troi 102	108.4095	12.091	3	0	1	0	0	0	0	0	3	3	0.0012	0.0014	0.0014	0.0017	0
5 Cong Troi 103	108.4667	11.9488	10	1	2	0.0003	0	0.0002	0	0.0003	11	12	0.0028	0.0034	0.0025	0.0030	0
Total Cong Troi			13	1	2	0.0002	0	0.0001	0	0.0002	12	14	0.0029	0.0034	0.0024	0.0029	0
6 Bidoup	108.6854	12.0475	10	2	3	0.0005	0.0012	0.0004	0.0009	0	10	9	0.0026	0.0031	0.0038	0.0045	0
Total			57	7	7	0.0012	0.0016	0.0003	0.0006	0.0001	16	23	0.0027	0.0033	0.0033	0.0039	0

Table S4 continued

				GSTG 1							*GSTH* 2						
Population	Longitude (°E)	Latitude (°N)	N	S	n_h	θ_w	θ_{ws}	π_t	π_s	π_a	S	n_h	θ_w	θ_{ws}	π_t	π_s	π_a
1 Da Chay 89A	108.6843	12.1758	9	3	3	0.0011	0.0015	0.0004	0.0006	0.0003	6	6	0.0035	0.0033	0.0051	0.0050	0.0056
2 Da Chay 90A	108.7015	12.1756	13	3	3	0.0010	0.0014	0.0009	0.0007	0.0010	7	7	0.0037	0.0037	0.0031	0.0028	0.0042
3 Da Chay 91B	108.6893	12.1938	12	3	4	0.0010	0.0028	0.0007	0.0019	0.0010	8	10	0.0043	0.0045	0.0044	0.0045	0.0044
Total Da Chay			34	7	7	0.0018	0.0033	0.0007	0.0010	0.0005	8	10	0.0034	0.0035	0.0043	0.0041	0.0047
4 Cong Troi 102	108.4095	12.091	3	0	1	0	0	0	0	0	4	5	0.0035	0.0037	0.0036	0.0035	0.0039
5 Cong Troi 103	108.4667	11.9488	10	3	3	0.0010	0.0015	0.0012	0.0015	0.0009	7	7	0.0040	0.0048	0.0059	0.0074	0.0019
Total Cong Troi			13	3	4	0.0010	0.0014	0.0012	0.0012	0.0010	7	7	0.0037	0.0044	0.0054	0.0067	0.0024
6 Bidoup	108.6854	12.0475	10	3	4	0.0010	0.0015	0.0005	0.0008	0.0002	6	9	0.0034	0.0040	0.0041	0.0043	0.0037
Total			57	8	10	0.0019	0.0035	0.0008	0.0011	0.0006	8	15	0.0030	0.0032	0.0046	0.0048	0.0040

Table S4 continued

				IFG 1934							*IFG* 8612						
Population	Longitude (°E)	Latitude (°N)	N	S	n_h	θ_w	θ_{ws}	π_t	π_s	π_a	S	n_h	θ_w	θ_{ws}	π_t	π_s	π_a
1 Da Chay 89A	108.6843	12.1758	9	2	3	0.0008	0.0031	0.0008	0.0033	0	4	5	0.0012	0.0014	0.0011	0.0013	0
2 Da Chay 90A	108.7015	12.1756	13	2	3	0.0007	0.0028	0.0008	0.0031	0	4	5	0.0011	0.0013	0.0012	0.0013	0
3 Da Chay 91B	108.6893	12.1938	12	2	4	0.0007	0.0028	0.0005	0.0020	0	4	5	0.0011	0.0013	0.0010	0.0011	0
Total Da Chay			34	2	4	0.0006	0.0022	0.0007	0.0028	0	8	9	0.0018	0.0020	0.0010	0.0013	0
4 Cong Troi 102	108.4095	12.091	3	1	2	0.0006	0.0023	0.0004	0.0018	0	1	2	0.0004	0.0005	0.0003	0.0004	0
5 Cong Troi 103	108.4667	11.9488	10	4	5	0.0015	0.0045	0.0009	0.0025	0.0003	4	4	0.0012	0.0010	0.0013	0.0013	0.0008
Total Cong Troi			13	4	5	0.0014	0.0042	0.0008	0.0023	0.0003	4	4	0.0011	0.0010	0.0012	0.0012	0.0007
6 Bidoup	108.6854	12.0475	10	2	4	0.0007	0.0030	0.0009	0.0035	0	7	8	0.0021	0.0024	0.0015	0.0018	0
Total			57	4	6	0.0010	0.0030	0.0007	0.0028	0.0001	11	11	0.0022	0.0023	0.0012	0.0013	0.0001

Table S4 continued

Population	Longitude (°E)	Latitude (°N)	N	*Rav* 2 S	n_h	θ_w	θ_{ws}	π_t	π_s	π_a	*SOS* 27 S	n_h	θ_w	θ_{ws}	π_t	π_s	π_a
1 Da Chay 89A	108.6843	12.1758	9	1	2	0.0004	0	0.0006	0	0.0010	1	3	0.0002	0.0001	0.0002	0.0002	0
2 Da Chay 90A	108.7015	12.1756	13	1	2	0.0003	0	0.0004	0	0.0007	0	1	0	0	0	0	0
3 Da Chay 91B	108.6893	12.1938	12	1	2	0.0004	0	0.0005	0	0.0007	2	3	0.0004	0.0001	0.0001	0.0002	0
Total Da Chay			34	1	2	0.0003	0	0.0005	0	0.0008	3	5	0.0005	0.0001	0.0001	0.0001	0
4 Cong Troi 102	108.4095	12.091	3	1	2	0.0006	0.0016	0.0004	0.0012	0	3	3	0.0011	0.0013	0.0013	0.0016	0
5 Cong Troi 103	108.4667	11.9488	10	1	2	0.0004	0	0.0007	0	0.0011	0	1	0	0	0	0	0
Total Cong Troi			13	2	3	0.0007	0.0009	0.0007	0.0003	0.0010	3	3	0.0006	0.0008	0.0004	0.0004	0
6 Bidoup	108.6854	12.0475	10	3	4	0.0011	0.0010	0.0008	0.0004	0.0011	0	1	0	0	0	0	0
Total			57	3	4	0.0007	0.0007	0.0006	0.0001	0.0009	5	6	0.0008	0.0011	0.0001	0.0002	0

Table S4 continued

Population	Longitude (°E)	Latitude (°N)	N	*TPP* 1 S	n_h	θ_w	θ_{ws}	π_t	π_s	π_a	*TPS* 4 S	n_h	θ_w	θ_{ws}	π_t	π_s	π_a
1 Da Chay 89A	108.6843	12.1758	9	0	1	0	0	0	0	0	1	2	0.0006	0	0.0004	0	0.0005
2 Da Chay 90A	108.7015	12.1756	13	1	2	0.0002	0	0.0001	0	0.0002	2	3	0.0010	0.0022	0.0004	0.0006	0.0004
3 Da Chay 91B	108.6893	12.1938	12	1	2	0.0002	0	0.0001	0	0.0002	3	4	0.0015	0.0044	0.0017	0.0031	0.0006
Total Da Chay			34	1	2	0.0002	0	0.0001	0	0.0002	3	4	0.0012	0.0034	0.0007	0.0014	0.0005
4 Cong Troi 102	108.4095	12.091	3	0	1	0	0	0	0	0	2	3	0.0017	0.0036	0.0013	0.0027	0.0008
5 Cong Troi 103	108.4667	11.9488	10	0	1	0	0	0	0	0	1	2	0.0005	0	0.0005	0	0.0002
Total Cong Troi			13	0	1	0	0	0	0	0	2	3	0.0010	0.0022	0.0007	0.0006	0.0007
6 Bidoup	108.6854	12.0475	10	1	2	0.0002	0.0003	0.0001	0.0001	0	1	2	0.0005	0	0.0002	0	0.0002
Total			57	2	3	0.0003	0.0002	0.0001	0	0.0001	3	4	0.0011	0.0031	0.0006	0.0010	0.0005

TableS5 Neutrality tests for individual nuclear loci as measured by Tajima's *D* , Fu and Li’s *D** and *F** , Fu's *Fs* , standardized Fay and Wu’s *H* , and the MK test.

	4CL							*CFX*						
						MK test							*MK* test	
Populations	Tajima's *D*	*D**	*F**	*Fs*	*H*	*Fisher*	*G* test	Tajima's *D*	*D**	*F**	*Fs*	*H*	*Fisher*	*G* test
1 Da Chay 90A	-1.7092	-2.2413	-2.4209	-1.2400	0.4298	0.5846	0.7960	0.7132	-0.1923	0.0974	-1.4480	0.5445	na	na
2 Da Chay 91B	-1.4953	-0.8560	-1.1998	-2.8420	-1.5134	1.0000	0.1440	0.4573	1.3732*	1.2829	0.1310	-0.8995	na	na
3 Da Chay 89A	na	na	na	na	na	na	na	0.9175	1.3827*	1.4451	0.9520	-0.9901	na	na
4 Cong Troi 102	na	na	na	na	na	na	na	1.1241	1.3958	1.4062	0.6150	-0.8514	na	na
5 Cong Troi 103	-0.5916	0.6495	0.3673	-0.0970	0.3992	1.0000	0	-0.3438	0.1161	-0.0191	-5.6180	-0.4155	na	na
6 Bidoup	-0.5278	-0.5935	-0.6606	-0.4640	0.5485	0.4965	na	1.6614	1.4100*	1.7172*	-0.6530	0.6158	na	na
Total	-1.7067	-1.4859	-1.8489	-5.3330	-1.4131	1.0000	0.1420	0.5462	0.0215	0.2579	-6.2870	0.1422	na	na

TableS5 continued

	GSTG 1							*GSTH* 2						
						MK test							*MK* test	
Populations	Tajima's *D*	*D**	*F**	*Fs*	*H*	*Fisher*	*G* test	Tajima's *D*	*D**	*F**	*Fs*	*H*	*Fisher*	*G* test
1 Da Chay 90A	-0.3026	0.9684	0.7035	0.6200	0.7313	0.5455	0.7540	-0.4630	-0.7341	-0.7607	-1.4830	0.0518	na	na
2 Da Chay 91B	-0.8023	0.9796	0.5541	-1.1950	0.4680	1.0000	na	0.0799	0.1405	0.1426	-3.4370	0.6814	na	na
3 Da Chay 89A	-1.7130	-2.3015	-2.4579	-1.0250	0.3137	1.0000	0.1030	1.4693	1.2590	1.5168	0.1800	0.7805	na	na
4 Cong Troi 102	-0.7093	-0.3748	-0.4688	0.0200	0.8514	1.0000	0.0170	0.1491	0.0713	0.0931	-2.3440	0.7020	na	na
5 Cong Troi 103	0.4212	1.0065	0.9732	1.1730	0.8311	1.0000	0.1030	1.5534	1.2998	1.5857	-0.0860	1.0810	na	na
6 Bidoup	-1.4407	-1.2550	-1.5017	-2.1350	0.4376	1.0000	0.1030	0.6711	0.5473	0.6723	-3.2180	0.9488	na	na
Total	-1.5137	-0.9882	-1.3912	-6.3180	0.3946	1.0000	0.0080	1.1898	1.2501	1.4582	-3.4510	0.9785	na	na

TableS5 continued

	IFG 1934							*IFG* 8612						
						MK test							*MK* test	
Populations	Tajima's *D*	*D**	*F**	*Fs*	*H*	*Fisher*	*G* test	Tajima's *D*	*D**	*F**	*Fs*	*H*	*Fisher*	*G* test
1 Da Chay 90A	0.2724	0.8256	0.7742	0.2990	-0.6822	0.14708	na	0.1359	0.0889	0.1185	-0.5400	0.7973	na	*na*
2 Da Chay 91B	-0.6072	0.8373	0.5053	-1.9790	-1.9773	0.14708	na	-0.3204	-0.8560	-0.8140	-1.0310	0.6068	na	na
3 Da Chay 89A	0.2204	0.8846	0.8103	0.1610	-0.7743	0.14708	na	-0.3022	-0.7011	-0.6807	-1.1430	0.4422	na	na
4 Cong Troi 102	-0.9330	-0.9502	-0.9647	-0.0030	0.5518	0.27273	na	-0.9330	-0.9502	-0.9647	-0.0030	-2.7588	na	na
5 Cong Troi 103	-1.2057	-0.7593	-1.0171	-2.2180	-0.9029	0.36230	1.5450	0.1862	-0.7593	-0.5732	0.4210	0.5099	na	na
6 Bidoup	0.4354	0.8662	0.8605	-0.9170	-0.3823	0.14708	na	-0.8673	0.6766	-0.6517	-1.9150	0.6827	na	na
Total	-0.4747	-0.2934	-0.4147	-2.0280	-0.8105	0.36230	1.5450	-1.1816	-1.9712	-2.0113	-4.2090	0.4113	na	na

TableS5 continued

	Rav 2						
						MK test	
Populations	Tajima's *D*	*D**	*F**	*Fs*	*H*	*Fisher*	*G* test
1 Da Chay 90A	0.3809	0.6121	0.6308	0.7840	0.6011	1.0000	na
2 Da Chay 91B	0.4803	0.6227	0.6704	0.8470	0.6138	1.0000	na
3 Da Chay 89A	1.1662	0.6669	0.9102	1.2150	0.5509	1.0000	na
4 Cong Troi 102	-0.9330	-0.9502	-0.9647	-0.0030	0.5518	na	na
5 Cong Troi 103	1.1513	0.6495	1.0100	1.4670	0.2245	1.0000	na
6 Bidoup	-0.6428	-1.2550	-1.2505	-1.0060	0.5405	0.4667	na
Total	-0.3515	-0.6153	-0.6244	-0.5640	0.4521	0.4667	na

	SOS 27						
						MK test	
Populations	Tajima's *D*	*D**	*F**	*Fs*	*H*	*Fisher*	*G* test
1 Da Chay 90A	na	na	na	na	na	na	*na*
2 Da Chay 91B	-1.5147	-2.1591	-2.2809	-2.0780	0.2574	na	na
3 Da Chay 89A	-1.5352	-1.9890	-2.1385	-1.7960	0.3260	na	na
4 Cong Troi 102	1.1241	1.3958	1.4062	0.6150	0.8514	na	na
5 Cong Troi 103	na	na	na	na	na	na	na
6 Bidoup	na	na	na	na	na	na	na
Total	-1.8627	-1.8098	-2.1612	-6.4980	0.1623	na	na

TableS5 continued

	TPP 1						
						MK test	
Populations	Tajima's *D*	*D**	*F**	*Fs*	*H*	*Fisher*	*G* test
1 Da Chay 90A	-1.1556	-1.6338	-1.7271	-1.0940	0.1803	0.4000	na
2 Da Chay 91B	-1.1593	-1.6058	-1.7042	-1.0280	0.1929	0.4000	na
3 Da Chay 89A	na	na	na	na	na	na	na
4 Cong Troi 102	na	na	na	na	na	na	na
5 Cong Troi 103	na	na	na	na	na	na	na
6 Bidoup	-1.1644	-1.5396	-1.6477	-0.8790	0.2245	0.4000	na
Total	-1.2920	-1.1017	-1.3595	-3.4760	0.0987	0.4000	na

	TPS 4						
						MK test	
Populations	Tajima's *D*	*D**	*F**	*Fs*	*H*	*Fisher*	*G* test
1 Da Chay 90A	-1.2239	-0.6891	-0.9657	-1.4990	0.3411	1.0000	0.1380
2 Da Chay 91B	-0.6322	-0.1889	-0.3614	-0.9820	0.6348	1.0000	0.6800
3 Da Chay 89A	-0.5290	0.6669	0.4045	-0.0110	0.4285	1.0000	na
4 Cong Troi 102	-1.1320	-1.1553	-1.1951	-0.8580	0.7341	1.0000	0.1380
5 Cong Troi 103	-0.0861	0.6495	0.5203	0.3810	0.5239	1.0000	na
6 Bidoup	-1.6439	-1.5396	-1.6477	-0.8790	0.2245	1.0000	na
Total	-0.7913	0.8159	0.3589	-1.4500	0.4252	1.0000	0.6800

*P<0.05; *na* , not caculated due to low polymorphism.

Table S6 Population differentiation (F_{ST}) for each region and total sample of *P. krempfii* based on individual and combined nuclear loci.

Regions	No. of populations	Locus										Multilocus
		IFG 8612	*CFX*	4*CL*	*TPP* 1	*IFG* 1934	*SOS* 27	*Rav* 2	*GSTG* 1	*GSTH* 2	*TPS* 4	
Group Da Chay	3	-0.016	0.074*	<0.001	-0.029	-0.005	0.023*	-0.016	0.051*	0.039	0.016	0.038*
Group Cong Troi	2	0.149	0.071	-0.043	Monomorphic	-0.027	0.471**	0.270*	-0.076	-0.001	-0.029	0.078*
Total range	6	0.013	0.085**	0.018	-0.019	0.002	0.204**	0.026	0.048*	0.043*	0.005	0.052**

*P < 0.05; **P < 0.01.

Table S7 Summary of nucleotide diversity and effective population size in 22 pine species based on nuclear genes. Species with limited range of distribution are in bold.

Species	No. of loci	L	N	π_t	π_s	θ_w	θ_{ws}	$N_e \times 10^4$*	Reference
Pinus krempfii	10	8950	57	0.0011	0.0015	0.0014	0.0020	1.43	This study
P. tabuliformis	7	3040	43	0.0085	0.0119	0.0107	0.0153	10.93	(Ma *et al.*, 2006)
	8	5763	48	0.0070	0.0150	0.0092	0.0173	12.37	(Gao *et al.*, 2012)
P. yunnanensis	7	3040	29	0.0067	0.0095	0.0055	0.0077	5.50	(Ma *et al.*, 2006)
	8	5763	40	0.0046	0.0101	0.0060	0.0101	7.21	(Gao *et al.*, 2012)
P. densata	7	3040	66	0.0086	0.0122	0.0101	0.0143	10.21	(Ma *et al.*, 2006)
	8	5763	136	0.0065	0.0138	0.0098	0.0153	10.93	(Gao *et al.*, 2012)
P. pinaster	11	9367	208	0.0055	0.0085	0.0062	na	6.07	(Eveno *et al.*, 2008)
	6	5588	122	0.0057	na	0.0045	na	3.20	(Grivet *et al.*, 2011)
	8	4746	14	0.0024	na	0.0021	na	1.52	(Pot *et al.*, 2005)
P. radiata	8	4746	23	0.0019	na	0.0019	na	1.36	(Pot *et al.*, 2005)
P. taeda	19	18027	32	0.0040	0.0064	0.0041	0.0066	4.70	(Brown *et al.*, 2004)
	28	na	32	na	na	0.0049	0.0059	4.20	(Neale & Savolainen, 2004)
	16	na	32	na	na	0.0046	0.0070	5.00	(Neale & Savolainen, 2004)
	18	10116	32	0.0051	0.0085	0.0053	0.0079	5.56	(Gonzalez-Martinez *et al.*, 2006)
	41	22652	32	0.0049	0.0070	0.0060	0.0093	6.6	(Ersoz *et al.*, 2010)
P. halepensis	10	5292	60	0.0018	na	0.0029	na	2.07	(Grivet *et al.*, 2009)
	6	4596	93	0.0031	na	0.0026	na	1.86	(Grivet *et al.* 2011)
P. sylvestris	16	6274	40	0.0034†	0.0070	0.0061†	0.0050	3.57	(Pyhäjärvi *et al.*, 2007)
	17	9880	40	0.0041	0.0057	na	0.0080	5.71	(Wachowiak *et al.*, 2011a)
	14	9635	40	0.0060	0.0077	na	0.0089	6.35	(Wachowiak *et al.*, 2009)
	11	30629	119	0.0032	0.0055	0.0053	0.0062	4.43	(Kujala & Savolainen, 2012)
	12	6670	40	0.0078	0.0117	na	0.0095	6.79	(Wachowiak *et al.*, 2011b)
	8	5417	28	0.0058	0.0076	0.0063	0.0055	3.94	(Ren *et al.*, 2012)
	8	21607	35	na	0.0079	na	0.010	7.14	(Pyhäjärvi *et al.*, 2011)
P. mugo	17	9909	12	0.0049	0.0067	na	0.0072	5.14	(Wachowiak *et al.*, 2011a)
	12	4851	169	0.0118	0.0185	na	0.0169	12.07	(Wachowiak *et al.*, 2013)
	310	119060	12	0.0077	0.0065	0.0076	0.0067	4.79	(Mosca *et al.*, 2012)
P. uncinata	12	4851	93	0.0113	0.0178	na	0.0134	9.57	(Wachowiak *et al.*, 2013)
P. cembra	280	109369	12	0.0018	0.0024	0.0019	0.0024	1.71	(Mosca *et al.*, 2012)
P. chiapensis	3	2800	7	0.0031	na	na	na	2.21	(Syring *et al.*, 2007)
P. ayacahuite	3	2800	8	0.0036	na	na	na	2.57	(Syring *et al.*, 2007)
P. monticola	3	2800	9	0.0092	na	na	na	6.57	(Syring *et al.*, 2007)
P. strobus	3	2800	10	0.0044	na	na	na	3.14	(Syring *et al.*, 2007)
P. thunbergii	15	5825	16	0.0061	na	0.00697	na	4.32	(Suharyanto & Shiraishi, 2011)
P. densiflora	15	5825	16	0.0053	na	0.00778	na	3.76	(Suharyanto & Shiraishi, 2011)
	8	5458	28	0.0060	0.0078	0.00642	0.0064	4.57	(Ren *et al.*, 2012)
P. luchuensis	15	5825	16	0.0050	na	0.00607	na	3.59	(Suharyanto & Shiraishi, 2011)
P. balfouriana	5	3089	40	0.0035	na	0.00325	na	2.32	(Eckert *et al.*, 2008)
P. canariensis	3	1327	384	0.0036	na	0.00367	na	2.56	(Lopez *et al.*, 2013)
P. contorta	21	9906	95	0.0025	0.0035	0.00251	0.0034	2.43	(Eckert *et al.*, 2012)

N, sample size; L, length in base pairs; π_t, nucleotide diversity based on total sites; π_s, nucleotide diversity based on silent sites; θ_w nucleotide polymorphism based on total sites; θ_w nucleotide polymorphism based on silent sites.
*N_e (effective population size) was calculated based on $\theta_{ws}/4\mu$ with the same assumptions as generation time of 50 years and mutation rate per year of 7×10^{-10} estimated for the genus *Pinus* by Willyard *et al.* (2007). If θ_w is not reported in original paper, then N_e is calculated sequentially based on π_s, θ_w or π_t.
†Calculated by Pavy *et al.* (2012) based on 14 of all 16 genes.
na, not reported in original paper.

Reference

Brown, G.R., Gill, G.P., Kuntz, R.J., Langley, C.H. & Neale, D.B. 2004. Nucleotide diversity and linkage disequilibrium in loblolly pine. *PNAS* **101:** 15255-15260.

Eckert, A.J., Tearse, B.R. & Hall, B.D. 2008. A phylogeographical analysis of the range disjunction for foxtail pine (*Pinus balfouriana*, Pinaceae): the role of Pleistocene glaciation. *Mol. Ecol.* **17:** 1983-1997.

Eckert, A.J., Shahi, H., Datwyler, S.L. & Neale, D.B. 2012. Spatially variable natural selection and the divergence between parapatric subspecies of lodgepole pine (*Pinus contorta*, Pinaceae). *Am. J. Bot.* **99:** 1323-1334.

Ersoz, E.S., Wright, M.H., Gonzalez-Martinez, S.C., Langley, C.H. & Neale, D.B. 2010. Evolution of disease response genes in loblolly pine: insights from candidate genes. *PloS One* **5:** e14234.

Eveno, E., Collada, C., Guevara, M.A., Leger, V., Soto, A., Diaz, L., Leger, P., Gonzalez-Martinez, S.C., Cervera, M.T., Plomion, C. & Garnier-Gere, P.H. 2008. Contrasting patterns of selection at *Pinus pinaster* Ait. drought stress candidate genes as revealed by genetic differentiation analyses. *Mol. Biol. Evol.* **25:** 417-437.

Gao, J., Wang, B., Mao, J.F., Ingvarsson, P., Zeng, Q.Y. & Wang, X.R. 2012. Demography and speciation history of the homoploid hybrid pine *Pinus densata* on the Tibetan Plateau. *Mol. Ecol.* **21:** 4811-4827.

Gonzalez-Martinez, S.C., Ersoz, E., Brown, G.R., Wheeler, N.C. & Neale, D.B. 2006. DNA sequence variation and selection of tag single-nucleotide polymorphisms at candidate genes for drought-stress response in *Pinus taeda* L. *Genetics* **172:** 1915-1926.

Grivet, D., Sebastiani, F., Gonzalez-Martinez, S.C. & Vendramin, G.G. 2009. Patterns of polymorphism resulting from long-range colonization in the Mediterranean conifer Aleppo pine. *New Phytol.* **184:** 1016-1028.

Grivet, D., Sebastiani, F., Alia, R., Bataillon, T., Torre, S., Zabal-Aguirre, M., Vendramin, G.G. & Gonzalez-Martinez, S.C. 2011. Molecular footprints of local adaptation in two Mediterranean conifers. *Mol. Biol. Evol.* **28:** 101-116.

Kujala, S.T. & Savolainen, O. 2012. Sequence variation patterns along a latitudinal cline in Scots pine (*Pinus sylvestris*): signs of clinal adaptation? *Tree Genet. Genomes* **8:** 1451-1467.

Lopez, R., de Heredia, U.L., Collada, C., Cano, F.J., Emerson, B.C., Cochard, H. & Gil, L. 2013. Vulnerability to cavitation, hydraulic efficiency, growth and survival in an insular pine (*Pinus canariensis*). *Ann. Bot.* **111:** 1167-1179.

Ma, X.F., Szmidt, A.E. & Wang, X.R. 2006. Genetic structure and evolutionary history of a diploid hybrid pine *Pinus densata* inferred from the nucleotide variation at seven gene loci. *Mol. Biol. Evol.* **23:** 807-816.

Mosca, E., Eckert, A.J., Liechty, J.D., Wegrzyn, J.L., La Porta, N., Vendramin, G.G. & Neale, D.B. 2012. Contrasting patterns of nucleotide diversity for four conifers of Alpine European forests. *Evol. Appl.* **5:** 762-775.

Neale, D.B. & Savolainen, O. 2004. Association genetics of complex traits in conifers. *Trends Plant Sci.* **9:** 325-330.

Pavy, N., Namroud, M.C., Gagnon, F., Isabel, N. & Bousquet, J. 2012. The heterogeneous levels of linkage disequilibrium in white spruce genes and comparative analysis with other conifers. *Heredity* **108:** 273-284.

Pot, D., McMillan, L., Echt, C., Le Provost, G., Garnier-Gere, P., Cato, S. & Plomion, C. 2005. Nucleotide variation in genes involved in wood formation in two pine species. *New Phytol.* **167:** 101-112.

Pyhäjärvi , T., Garcia-Gil, M.R., Knürr, T., Mikkonen, M., Wachowiak, W. & Savolainen, O. 2007. Demographic history has influenced nucleotide diversity in European *Pinus sylvestris* populations. *Genetics* **177:** 1713-1724.

Pyhäjärvi, T., Kujala, S.T. & Savolainen, O. 2011. Revisiting protein heterozygosity in plants-nucleotide diversity in allozyme coding genes of conifer *Pinus sylvestris*. *Tree Genet. Genomes* **7:** 385-397.

Ren, G.P., Abbott, R.J., Zhou, Y.F., Zhang, L.R., Peng, Y.L. & Liu, J.Q. 2012. Genetic divergence, range expansion and possible homoploid hybrid speciation among pine species in Northeast China. *Heredity* **108:** 552-562.

Suharyanto & Shiraishi, S. 2011. Nucleotide diversities and genetic relationship in the three Japanese pine species; *Pinus thunbergii*, *Pinus densiflora,* and *Pinus luchuensis*. *Diversity* **3:** 121-135.

Syring, J., Del Castillo, R.F., Cronn, R. & Liston, A. 2007. Multiple nuclear loci reveal the distinctiveness of the threatened, neotropical *Pinus chiapensis*. *Syst. Bot.* **32:** 703-717.

Wachowiak, W., Balk, P.A. & Savolainen, O. 2009. Search for nucleotide diversity patterns of local adaptation in dehydrins and other cold-related candidate genes in Scots pine (*Pinus sylvestris* L.). *Tree Genet. Genomes* **5:** 117-132.

Wachowiak, W., Palme, A.E. & Savolainen, O. 2011a. Speciation history of three closely related pines *Pinus mugo* (T.), *P. uliginosa* (N.) and *P. sylvestris* (L.). *Mol. Ecol.* **20:** 1729-1743.

Wachowiak, W., Salmela, M.J., Ennos, R.A., Iason, G. & Cavers, S. 2011b. High genetic diversity at the extreme range edge: nucleotide variation at nuclear loci in Scots pine (*Pinus sylvestris* L.) in Scotland. *Heredity* **106:** 775-787.

Wachowiak, W., Boratynska, K. & Cavers, S. 2013. Geographical patterns of nucleotide diversity and population differentiation in three closely related European pine species in the *Pinus mugo* complex. *Bot. J. Linn. Soc.* **172:** 225-238.

Willyard, A., Syring, J., Gernandt, D.S., Liston, A. & Cronn, R. 2007. Fossil calibration of molecular divergence infers a moderate mutation rate and recent radiations for *Pinus*. *Mol. Biol. Evol.* **24:** 90-101.

Fig. S1

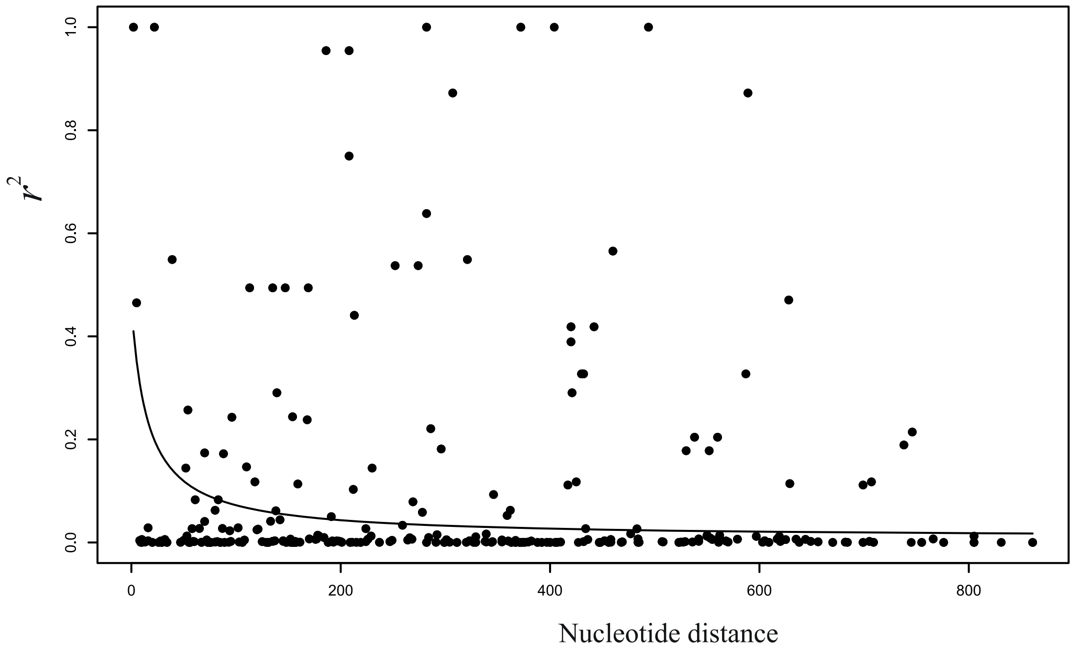

r²
1.0
0.8
0.6
0.4
0.2
0.0
0
200
400
600
800
Nucleotide distance

Fig. S2

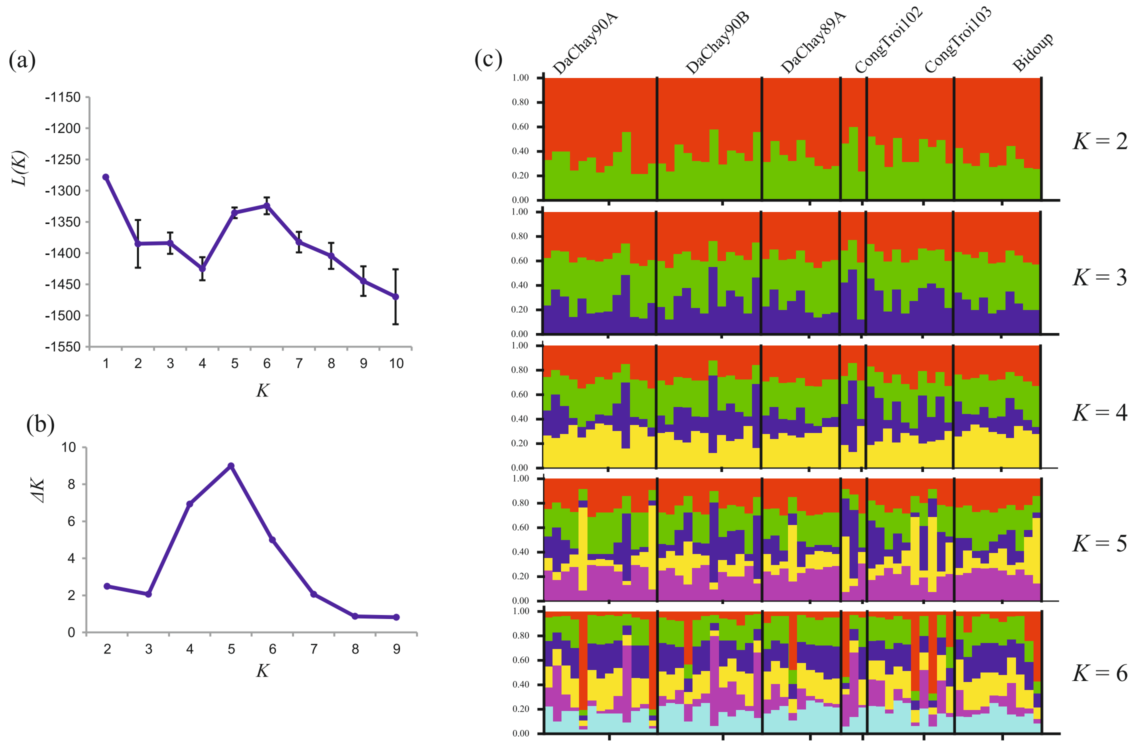

(a)
L(K)
-1150
-1200
-1250
-1300
-1350
-1400
-1450
-1500
-1550
1 2 3 4 5 6 7 8 9 10
K
(b)
ΔK
10
8
6
4
2
0
2 3 4 5 6 7 8 9
K
(c)
DaChay90A
DaChay90B
DaChay89A
CongTroi102
CongTroi103
Bidoup
1.00
0.80
0.60
0.40
0.20
0.00
K = 2
K = 3
K = 4
K = 5
K = 6

Fig. S3

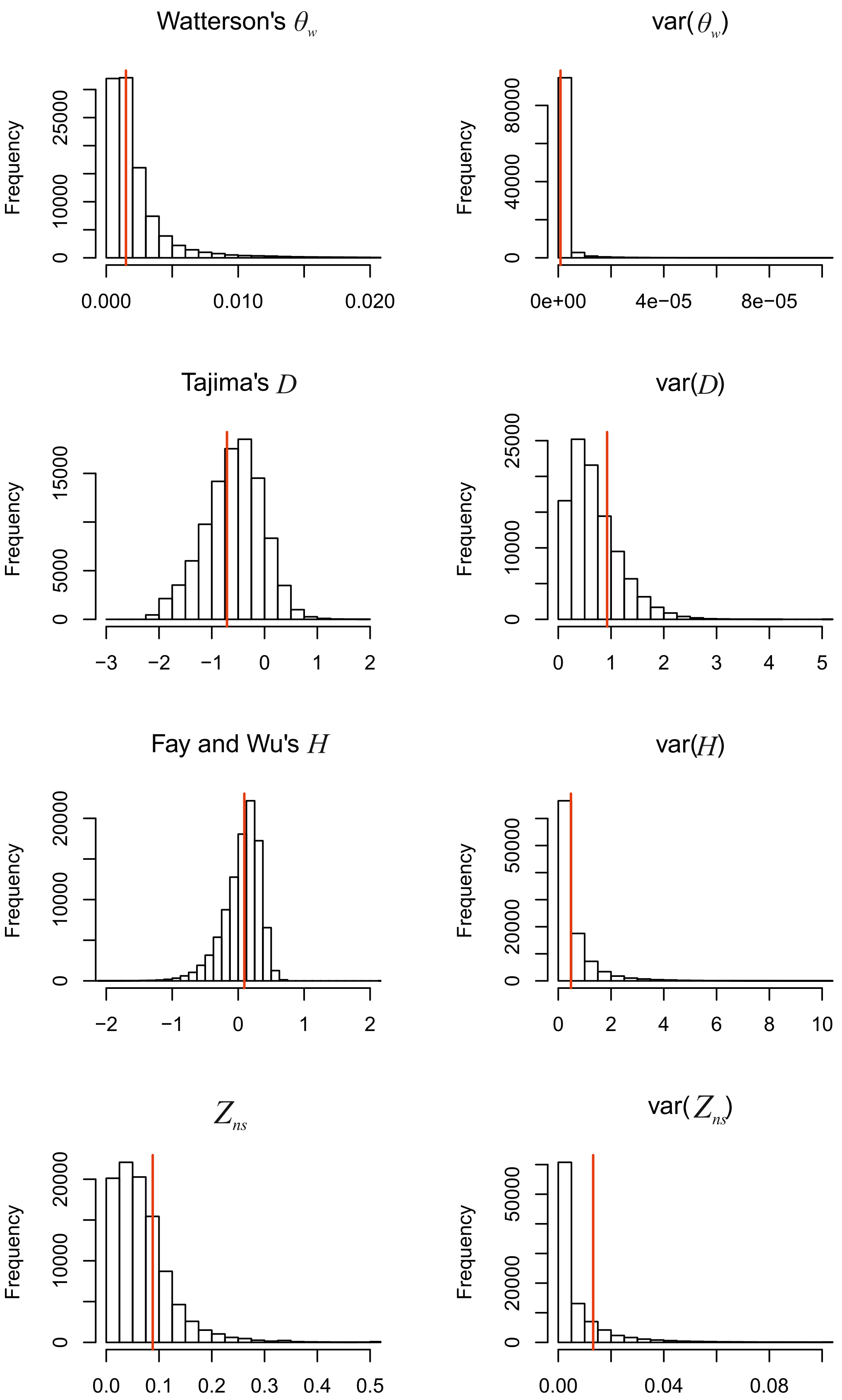
Watterson's θ_w
Frequency
0
10000
25000
0.000
0.010
0.020
var(θ_w)
Frequency
0
40000
80000
0e+00
4e−05
8e−05
Tajima's D
Frequency
0
5000
15000
−3
−2
−1
0
1
2
var(D)
Frequency
0
10000
25000
0
1
2
3
4
5
Fay and Wu's H
Frequency
0
10000
20000
−2
−1
0
1
2
var(H)
Frequency
0
20000
50000
0
2
4
6
8
10
Z_{ns}
Frequency
0
10000
20000
0.0
0.1
0.2
0.3
0.4
0.5
var(Z_{ns})
Frequency
0
20000
50000
0.00
0.04
0.08
